# Gonadal steroid levels in rock pigeon eggs do not represent adequately maternal allocation

**DOI:** 10.1038/s41598-018-29478-4

**Published:** 2018-07-25

**Authors:** Neeraj Kumar, Martijn van Faassen, Bonnie de Vries, Ido Kema, Manfred Gahr, Ton G. G. Groothuis

**Affiliations:** 1 Behavioural Biology, Groningen Institute for Evolutionary Life Sciences, University of Groningen, Groningen, The Netherlands; 2 Laboratory Medicine, University Medical Center Groningen, University of Groningen, Groningen, The Netherlands; 3 Behavioural Neurobiology, Max Planck Institute for Ornithology, Seewiesen, Germany

## Abstract

Maternal hormones deposited in the egg can provide a powerful model for the study of maternal effects. The differential amount of maternal hormones in the yolk of freshly laid eggs is assumed to represent differential maternal allocation. However, some evidence suggests that these amounts do not reflect maternal allocation that in fact takes place before ovulation. We compared the amounts of a wide array of gonadal steroids and their metabolites in the yolk of pre-ovulatory follicles with those of freshly laid eggs of rock pigeons using mass spectrometry. We found that between the follicle and egg stages the levels of progesterone increase whereas androstenedione and testosterone decrease in which the strength of decrease was dependent on the laying order of the egg. For conjugated estrone the change between follicle and egg differed in direction for first and second laying position yielding a significant interaction effect. For conjugated testosterone the interaction did not reach but was close to significance. This extremely early steroid metabolism was not due to maternal enzymes in the yolk as indicated by incubation of pre-ovulatory yolks treated with proteinase-K, a protein digesting enzyme. The results have significant consequences for the functional and evolutionary interpretation as well as experimental manipulation of hormone-mediated maternal effects.

## Introduction

Maternal hormone deposition in eggs of oviparous species is recognized as a maternal tool to adjust offspring phenotype to the current or future environment of the offspring^1–4^. Most studies on maternal hormones have been performed with bird species as their embryos develop outside the mother’s body in a sealed environment facilitating measurements and manipulation of maternal hormone deposition while their ecology and evolution are well known. Maternal hormone concentrations in the eggs, mostly measured in yolks, show clear and systematic variation among species, females of the same species, nests of the same mother, and eggs of the same nest. Effects of these yolk hormones have been demonstrated on physiology, morphology, and behaviour of the offspring (reviewed in^4^).

The variation in yolk hormone levels is assumed to be due to differential maternal hormone deposition depending on some context, for instance the laying order of eggs, mate quality, social hierarchy of the female within a group, seasonal variation in predation risk, food availability, etc. Yolk hormone levels are often measured at the time of oviposition or a few days later. However, the yolk formation and hormone addition to the yolk is finished by the time of ovulation, as no more yolk can be added to the egg after ovulation. Intriguingly, some studies show that the concentrations of androgens decline significantly already between the time of ovulation and oviposition^5,6^ and one study showed an increase in progesterone between pre- and post-ovulatory follicles^7^. However, the cause and consequence of this different pattern of change among different classes of steroids are unknown.

The above-mentioned decline in some yolk hormone concentrations between the time of ovulation and oviposition could potentially be explained by (1) simply the addition of albumen and water, diluting the maternal deposited hormone concentrations, or (2) very early metabolic processes. Such metabolic processes have been found after oviposition by developing embryos upon egg incubation^8–11^ but whether this occurs already before oviposition in the reproductive track of the mother has not yet been tested. The enzymes for this could then be deposited by the mother in the egg or the enzymes could already be from embryonic origin. We tested the two hypotheses with the following experiments and predictions. We analysed total amounts of the hormones both in follicles and in the complete egg at oviposition. If the early decrease in hormone concentrations before oviposition is due to dilution with albumen and water, the total amount of hormones deposited in the ovarian follicle should be the same as in the whole egg (yolk plus albumen) at oviposition, only its concentration should decrease. If, however, this is not the case and the total amounts of hormones decrease, that would indicate hormone metabolism. In addition we analysed the potential decline in maternally deposited hormones by adding proteinase-K, a protein digesting enzyme, to the follicle yolk. If this would block the decline in maternally deposited hormones it would confirm a role for enzymatic processes deposited by the mother in the yolk.

**Figure 1:**
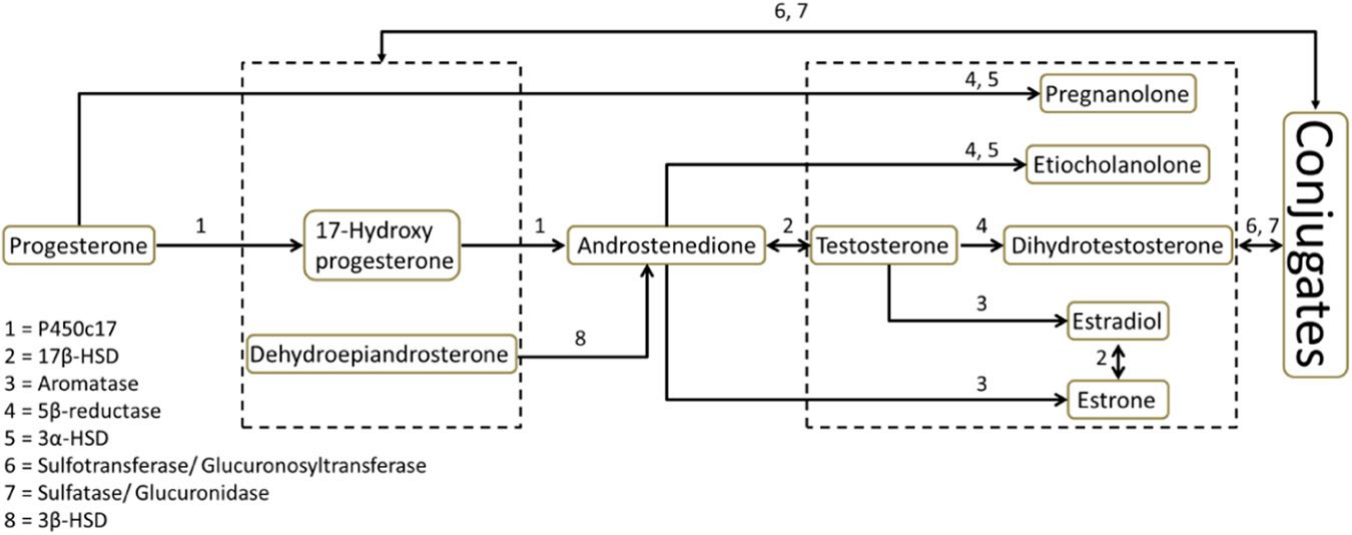
The gonadal steroid metabolic pathway of the analysed compounds including 10 free and 8 conjugated forms. Only the compounds within the dashed boxes can be conjugated. Numbers represent the enlisted enzymes involved in the pathway with the following abbreviations- P450c17: steroid 17 alpha-hydroxylase/17,20 lyase; HSD: hydroxysteroid dehydrogenase.

Since other studies have found hormone metabolism after oviposition (see above), we anticipated that such metabolism would also occur before oviposition, raising the question which hormones are converted to which metabolites and to which extent. Therefore we compared the hormone levels of 18 targeted hormones (10 free, 8 conjugated) of the steroid metabolic pathway of gonadal hormones (Fig. 1) at both the pre-ovulatory follicle and oviposition stage in eggs of the rock pigeon (*Columba livia*). The advantage of using this species is that they lay two eggs per nest and at oviposition the second eggs contain systematically higher levels of yolk androgens as compared to the first eggs^12^. This allowed us to test whether the differences in eggs based on the laying order remain the same at ovulation as at oviposition, the latter being the most commonly used time point for estimating maternal hormone allocation. For identification and quantification of hormones, liquid and gas chromatography combined with tandem mass spectrometry was used. Measuring all the components of the pathway shown in Fig. 1 also made it possible to monitor the metabolic outcomes. Furthermore, we also investigated the role of maternal enzymes in the yolk for the postulated early steroid metabolism by incubating follicle yolks treated with Proteinase-K that digests the maternal protein enzymes.

## Results

**Figure 2:**
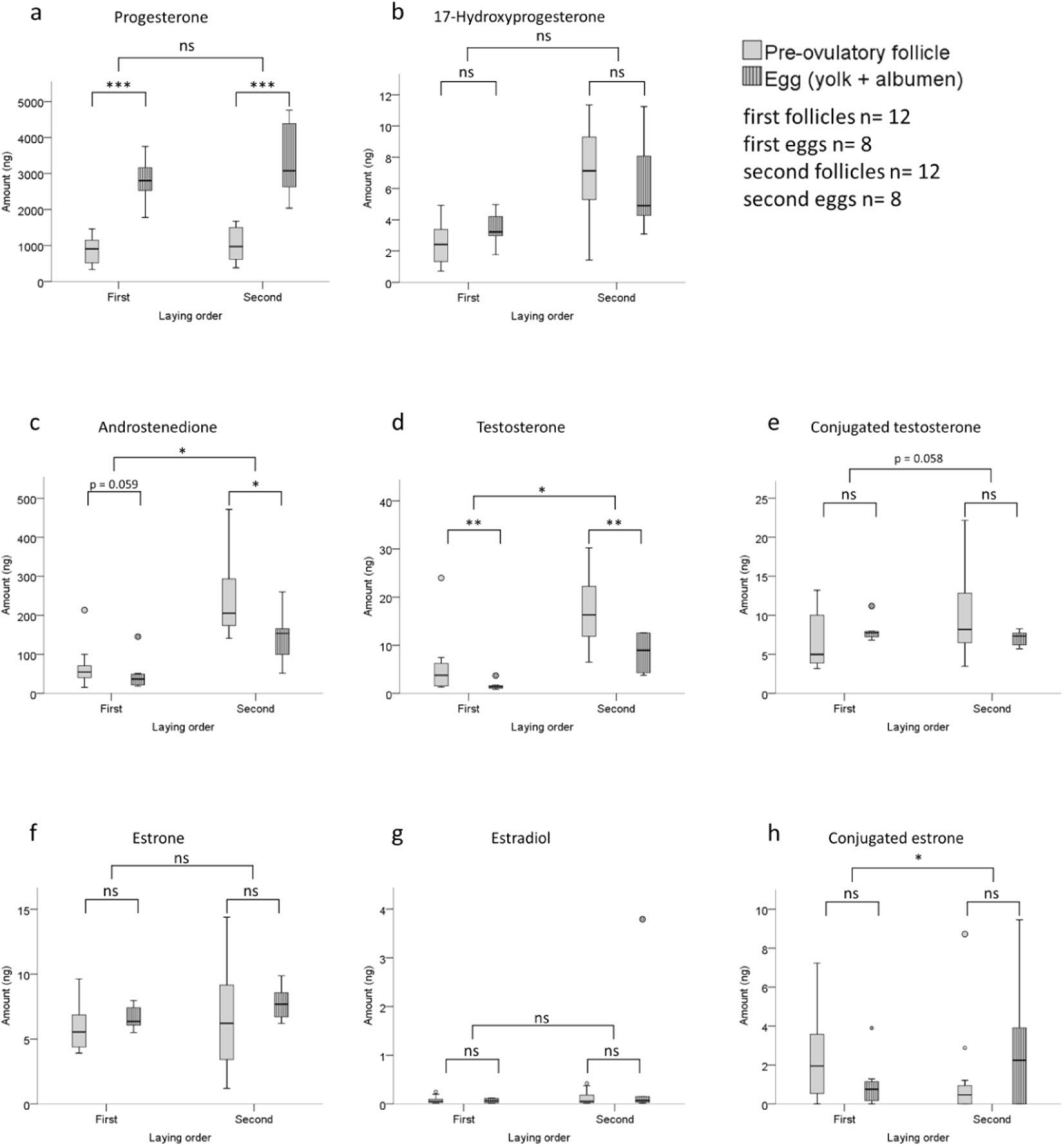
The difference in the amount of detectable gonadal steroid hormones in yolks of mature pre-ovulatory follicles compared with eggs (yolk plus albumen) for first and second eggs of the rock pigeon. The boxplots represent 25th and 75th percentiles as lower and upper boundaries of the box with median values as central tendencies, and the error bars represent 1.5 times of the interquartile range. *p < 0.05; **p < 0.01; ***p < 0.001; ns – not significant.

The results comparing follicles (yolk of mature pre-ovulatory follicles) and eggs (yolk plus albumen) for the hormones that were detectable are shown in Fig. 2, with the statistical parameters shown in Table 1. The levels of progesterone increase similarly for both first and second eggs as compared to the levels in first and second follicles respectively (p < 0.001, Table 1, Fig. 2a). There is no change in the levels of 17-hydroxyprogesterone between follicle and egg for each laying order (Fig. 2b). The levels of androstenedione and testosterone decrease between follicle and egg stage for each laying order with a significant interaction (p < 0.05, Table 1, Fig. 2c,d), with the decline for the second laying order being larger than for the first laying order. For conjugated testosterone there is a non-significant increase between follicle and egg for the first laying order and a non-significant decrease for the second laying order (Fig. 2e), leading to an interaction close to significance (p = 0.058). There is no change in the levels of estrone and estradiol (Fig. 2f,g). The pattern of change between follicle and egg for conjugated estrone follows the opposite trend for first and second laying order: although not significantly, the levels tend to decrease for the first laying order, and increase for the second laying order, with a significant interaction (p < 0.05, Table 1, Fig. 2h). The levels of etiocholanolone were below the quantification limits (1.0 nmol/L) for most of the samples except a few follicle samples, and thus excluded from statistical analysis. However, it suggests that the levels of etiocholanolone are either remaining the same or decline between ovulation and oviposition. Table 1 summarizes the relative effect (the standardized effect size in terms of coefficients (beta) from the statistical model) of time (pre-ovulatory follicle versus egg) for each hormone.

**Table 1:** Statistical parameters corresponding to Fig. 2.

Steroid	Statistical parameters	Time (pre-ovulatory follicle vs egg)		Interaction
		for laying order first	for laying order second	
Progesterone	Coefficient (beta)	1.585	1.897	−0.312
	Std Error	0.175	0.316	0.362
	p value	**<0.001**	**<0.001**	0.393
17-Hydroxyprogesterone	Coefficient (beta)	0.323	−0.315	0.638
	Std Error	0.183	0.458	0.493
	p value	0.093	0.502	0.204
Androstenedione	Coefficient (beta)	−0.170	−0.925	0.755
	Std Error	0.087	0.332	0.343
	p value	0.059	**0.015**	**0.035**
Testosterone	Coefficient (beta)	−0.297	−1.103	0.806
	Std Error	0.092	0.321	0.334
	p value	**0.005**	**0.003**	**0.021**
Estrone	Coefficient (beta)	0.298	0.529	−0.231
	Std Error	0.266	0.468	0.538
	p value	0.263	0.288	0.672
Estradiol	Coefficient (beta)	−0.139	−0.539	0.400
	Std Error	0.290	0.483	0.563
	p value	0.626	0.308	0.519
Conjugated testosterone	Coefficient (beta)	0.220	−0.750	0.970
	Std Error	0.309	0.386	0.495
	p value	0.461	0.081	0.058
Conjugated estrone	Coefficient (beta)	−0.667	0.925	−1.592
	Std Error	0.389	0.546	0.670
	p value	0.097	0.117	**0.023**

**Figure 3:**
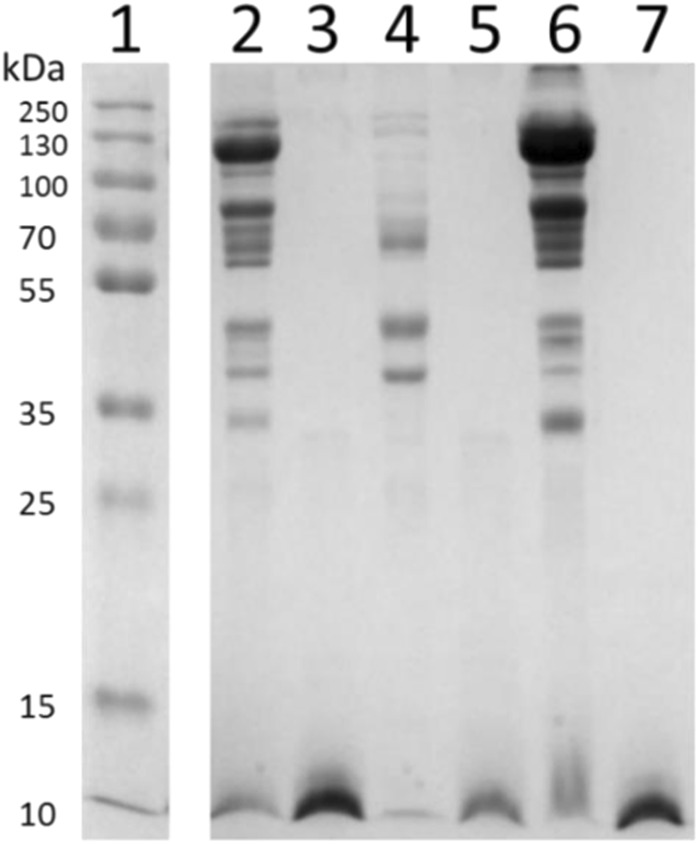
Digestion of yolk proteins with proteinase-K at a final concentration of 2.0 mg/ml for 3 hours at 37 °C as visualized on 12% Tris-Glycine gel. Lane 1 shows bands of a commercial protein molecular weight marker (ThermoFisher Scientific). Yolk proteins are shown before (lanes 2, 4, 6) and after (lanes 3, 5, 7) proteinase-K treatment for three fractions – crude (lanes 2 and 3), supernatant (lanes 4 and 5), and pellet (lanes 6 and 7). Lanes 1 and 2–7 are cropped from the same gel which is provided in Fig. S1 in the supplementary information.

**Figure 4:**
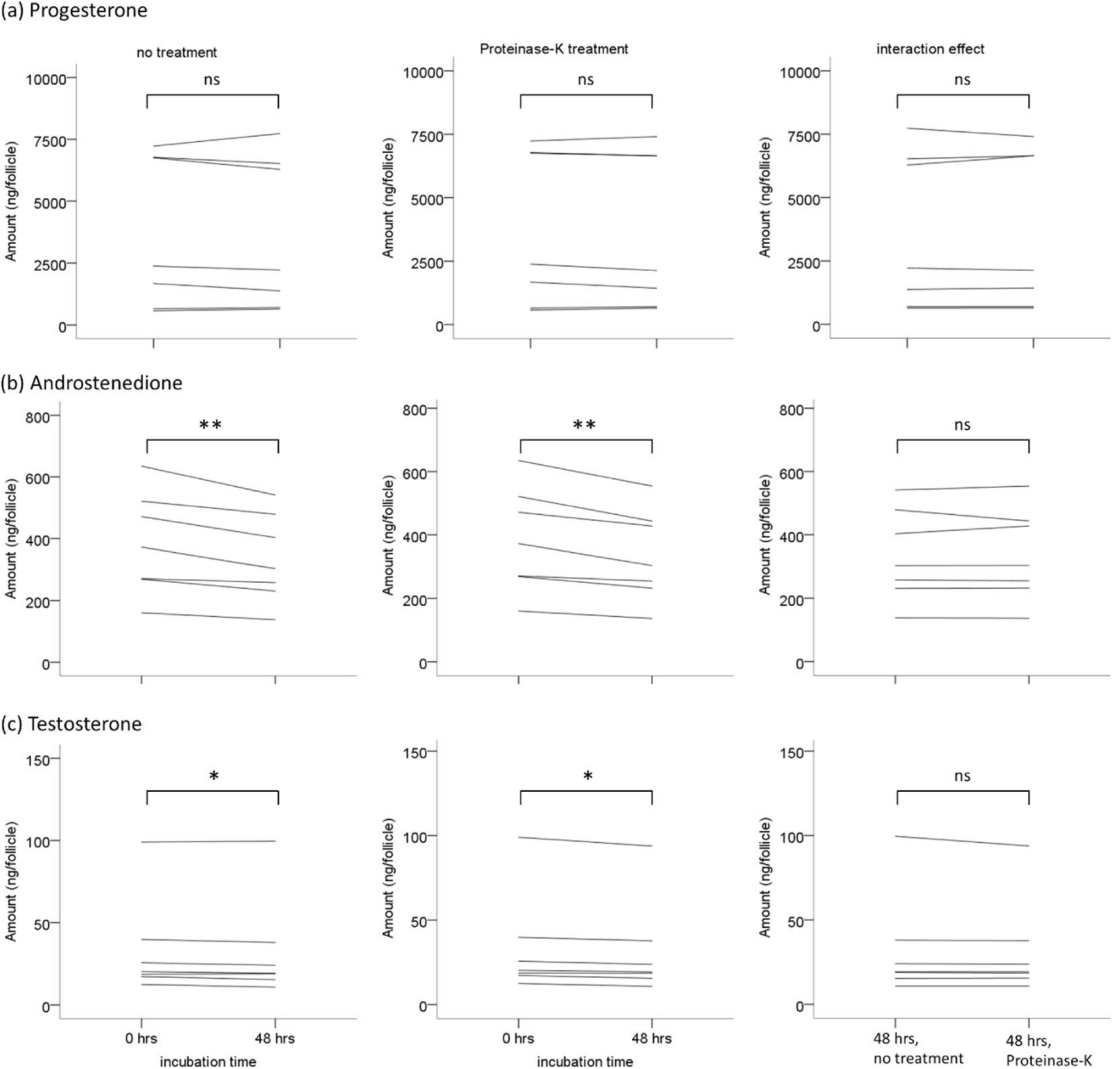
Effect of incubation duration, equivalent to the time difference between ovulation and oviposition on follicular yolk hormone levels with and without Proteinase-K treatment prior to incubation. n = 7; *p < 0.05; **p < 0.01.

Figure 3 shows the digestion of maternal enzymes in the yolk by Proteinase-K, indicating that the treatment indeed breaks down proteins. The levels of androstenedione and testosterone decline significantly (p < 0.01 and <0.05 respectively; Fig. 4) when follicular yolks are incubated for 48 hours at 41.4 °C, which mimics the internal body temperature and time difference between ovulation and oviposition in rock pigeons. However, when follicular yolks were treated with proteinase-K prior to incubation, and thus devoid of any activity of maternal enzymes, the levels of androstenedione and testosterone still declined (p < 0.01 and p < 0.05 respectively). This suggests that the decline is not due to maternal yolk enzymes. However, in terms of effect size the level of decline is much smaller with incubation of yolks alone than observed in natural egg development in maternal reproductive tract with the addition of albumen and egg shell (Supplementary Tables 2–3), indicating involvement of maternal (other than deposited in the yolk) and/or embryonic enzymatic processes instead of simply non-enzymatic degradation of hormones. There was no effect of maternal enzymes in the yolk on progesterone levels.

## Discussion

Hormone-mediated maternal effects have received a lot of attention in the past two decades, with the majority of studies having used bird species. By studying changes in gonadal hormones in mature first and second follicles and first and second freshly laid eggs of rock pigeons and by using a treatment aiming at removing action of steroidogenic enzymes in the yolk, we aimed to answer the following questions: (1) Do hormone levels at oviposition indeed indicate maternal hormone allocation or do these levels change between ovulation (directly after hormones are deposited by the mother in the yolk) and oviposition that takes place 48 hours later? (2) If there is a change, which hormones are converted to which other hormones? (3) Is this caused by maternal yolk enzymes; (4) Is this change similar or different for eggs of a different laying order. The results can have consequences for the interpretation of maternal hormone allocation, the role of the embryo and parent-offspring conflict, and experimental manipulation of egg hormones.

Earlier we reported the laying order difference for androgens in rock pigeon eggs at oviposition using radioimmunoassays^12^. Here we confirmed these findings using LC-MS/MS and found a laying order difference in the levels of 17-hydroxyprogesterone too (Supplementary Table 4).

The levels of progesterone increased whereas androstenedione and testosterone decreased over egg development between ovulation and oviposition (Fig. 2a,c,d). This indicates that concentrations of these hormones in eggs at oviposition may not reflect maternal allocation (see below). There was no change in the levels of estrone and estradiol (Fig. 2f,g). Levels of estradiol were extremely low (0.02–0.42 ng in entire follicle/egg; 0.003–0.177 pg/mg) as compared to other steroids. Further downstream metabolites- dihydrotestosterone (5α or 5β), etiocholanolone, and pregnanolone were not detectable, except etiocholanolone in some follicles. Therefore, the decline in androstenedione and testosterone levels cannot be explained by their conversion to their downstream metabolites (see Fig. 1). One possibility is that androgens are converted back to progesterone in bird eggs through a yet undiscovered metabolic pathway, or there are other, not yet detected metabolites. As we have analysed the known gonadal steroids with well-known biological functions as well as their known metabolites it is likely that other metabolites will have no important biological function, but this requires further research.

Bowden *et al*.^7^ previously found an increase in yolk progesterone concentration between pre- and post-ovulatory follicles in a turtle species. We found that the amount of progesterone in freshly laid bird eggs was significantly higher than even the mature follicles. In contrast to the Bowden study, we found a decrease in androgens, the primary hormones that are very frequently measured in the large literature on hormone mediated maternal effects in birds, but the extent of the decline differs based on egg laying order. Our data indicate that the levels of the most frequently analysed hormones in freshly laid eggs, testosterone, androstenedione, and progesterone, cannot be accurately used as indicator of maternal allocation. This is because the actual maternal allocation in the follicle stage can be different both in absolute levels as well as in indicating differences between laying order.

Conjugation of active steroids has been proposed as a mechanism by which the embryo could regulate its exposure to maternal hormones^13^. Out of the eight conjugated steroids that we investigated (see Fig. 1), only two were detectable: conjugated testosterone and conjugated estrone. Clairardin *et al*.^14^, and Paitz and Bowden^15^ have suggested conjugation of estrone as a potential pathway for estrogen metabolism in a turtle species, with its relevance to environmental endocrine disruptors. We found a trend for a decrease in the levels of conjugated estrone in first eggs as compared to first follicles, whereas an increase in second eggs. There was no change in conjugated testosterone in first eggs and a non-significant decrease in second eggs. Therefore, steroid conjugation cannot explain our observed change in steroid levels between follicle formation and oviposition. The free as well as conjugated dehydroepiandrosterone were below the quantification limit (2.0 nmol/L), suggesting that the steroidogenic pathway in bird eggs mainly involves 17-hydroxyprogesterone (see Fig. 1).

The increase of progesterone in both the first and second eggs (as compared to respective follicles) could either be due to de novo synthesis from its precursors (cholesterol and/or pregnenolone) in the yolk, or due to conversion of androgens to progesterone as already suggested above.

Since the decline in androstenedione and testosterone was found in the entire yolk-albumen homogenates, this excludes the possibility of simple migration of hormones from yolk to albumen or dilution due to mixing of yolk and albumen during incubation^16^, and rather suggests metabolism too. We excluded the possibility of metabolism due to maternal enzymes in the yolk as there is no difference in the decline of androstenedione and testosterone or increase in progesterone when follicular yolks are incubated with or without the activity of maternal enzymes in the yolk (Fig. 4). It remains unknown whether this early metabolism is due to embryonic enzymes including those present in the extra-embryonic membranes^17^, or maternal enzymes in the egg other than in the yolk, such as in yolk membrane, albumen, eggshell membrane, or eggshell, as suggested by Paitz and Bowden^15^ for turtle eggs.

The rate of decline in androstenedione and testosterone amounts is different between first and second eggs, i.e. there is a significant interaction between the egg laying order and time of egg development (Fig. 2c,d). The interaction was also close to significance for conjugated testosterone (p = 0.058, Fig. 2e), and significant for conjugated estrone (Fig. 2h). It might be that the first and second embryos possess different metabolic capacity for conjugation already during early developmental phases. Alternatively, the mother might be depositing different amount of enzymes involved in conjugation and/or deconjugation.

In an interesting similar study on domestic quail^18^ no change in total amount of testosterone was found between ovulation and oviposition. There are two differences between this study and ours: first, the method of hormone determination is different, being radioimmunoassay in the Okuliarova study whereas more specific and reliable mass spectrometry^19–21^ in ours. Second, the Okuliarova study used the domestic quail, a highly domesticated species that is selected to lay eggs every day instead of well-defined clutches with a limited number of eggs like in rock pigeon and wild birds in general. This calls for more comparative studies on hormone dynamics in developing eggs in order to establish how general the patterns are that we report in this study.

Hormone injections into the egg have revealed inconsistency in the magnitude and direction of hormone-mediated maternal effects^4^. Differences in early metabolism of hormones between species, egg position, or ecological context may potentially explain these differences. Moreover, it may reveal the extent of parent-offspring conflict as suggested by several authors (see above). If the early steroid metabolism found in this study was due to the embryonic activity, then our results indicate a differential role of the embryo based on its laying order already before the egg laying as in theory the maternal allocation and embryonic metabolism of hormones could work antagonistically in different ecological contexts^2^. The role of the embryo can be simply analysed by repeating the study with unfertilized eggs.

Our results do pose a potential problem for further research in this field as hormone amounts in freshly laid eggs do not adequately reflect maternal allocation. Ideally, one would first make for each species under study a comparison between hormone amounts in follicles and freshly laid eggs to calibrate further studies on the latter. A first step would be to do at least a few of such studies to clarify how general our conclusions are. In addition, methodology can be developed for assessing via biopsies and manipulating hormones directly in the follicles by applying small surgery that is used in field studies to assess the developmental stage of the gonads or by using ultrasound for taking biopsies from the yolk at the right place without the need for major surgery. Such approaches may help the field also in solving the problem of inconsistencies in the literature concerning effects of maternal hormones in the experimental studies on birds, a better understanding of eco-evolutionary effects and mother-offspring conflict. Finally, it should be realized that our findings have no bearing on the results of experimental manipulation of hormones in the egg and that the differences in hormone amount between follicles and freshly laid eggs are mostly quantitative and not qualitative, certainly for eggs within the same laying position.

In conclusion, our findings that maternal hormones in bird eggs are metabolized already between ovulation and oviposition, and that the rate and direction of metabolism differ between eggs that differ in their position within the laying order, adds a new layer of complexity to the understanding of hormone mediated maternal effects and makes it clear that the estimates of maternal allocation from oviposition are inadequate. Since we do not know whether this early metabolism is maternal or embryonic, we cannot yet correctly interpret the role of the mother and of the offspring in this process and thereby the role of parent-offspring conflicts in hormone mediated maternal effects. Moreover, the demonstration of differential early metabolism of egg hormones before oviposition may explain discrepancies in the literature on the effect of hormone injections into the egg. These new results do not make the field of hormone mediated maternal effects in egg laying species obsolete: our results still demonstrate systematic differences in hormone amounts between eggs at oviposition, and hormone injections in eggs have convincingly demonstrated that egg androgens have a wide array of effects on the embryo, and chick and even into adulthood^4^. But the study does demonstrate the relevance of mechanistic approaches for the functional and evolutionary interpretation of biological phenomena.

## Methods

### Animal housing

All animal procedures were carried out at the animal facility of the University of Groningen. The procedures were in accordance with the relevant guidelines and regulations of the animal welfare committee of the University of Groningen and were approved by the committee under license 6835B. Breeding stock was housed in outdoor aviaries under natural light and temperature conditions, *ad libitum* access to food (a mixture of commercial pigeon seeds (Kasper Faunafood, product 6721 and 6712), P40 vitamin supplement (Kasper Faunafood, product P40), and small stones or grit), fresh water, and nest boxes (60 cm × 50 cm × 36 cm) with breeding bowls and nesting material. Each bird had a unique combination of coloured leg bands for identification. Daily observations were made for food and water availability, pair formation, nest building, and egg laying.

### Follicle and egg collection

Follicles and eggs were collected from separate females. For egg collection nests were checked twice daily for egg laying and both first and second freshly laid eggs of a clutch were used for hormone analyses. Follicles were dissected after euthanasia using carbon dioxide from a separate group of females. As it required killing of the females to collect follicles, it was not possible to collect both first and second mature follicles from the same females, hence separate females were used. Time of production of first follicles was estimated based on laying intervals between clutches. As this was hampered by variation in inter-clutch intervals (6–15 days) we increased our sample size by using follicles from both adult females which had hatched from control eggs and testosterone injected eggs as part of another study, which had no effect at all on the statistical outcomes. The follicles were weighed immediately and stored at −20 °C until hormone extractions took place. Weight-matched first (n = 12) and second follicles (n = 12) were used for comparing steroid levels between follicles and that with first (n = 8) and second eggs (n = 8).

### Extraction and mass spectrometric analyses of steroids

We targeted 10 free steroids – progesterone, 17-hydroxyprogesterone, dehydroepiandrosterone, androstenedione, testosterone, pregnanolone, etiocholanolone, dihydrotestosterone, estradiol, and estrone. We also analysed their conjugated form as all of them can form conjugates except progesterone and androstenedione (as they lack a free hydroxyl group in their structure). Thus, in total we analysed 18 compounds (Fig. 1). However, the following 10 compounds were below their quantification limits in all samples: (a) the free forms of dehydroepiandrosterone, pregnanolone, etiocholanolone, and dihydrotestosterone, (b) the conjugated compounds of 17-hydroxyprogesterone, dehydroepiandrosterone, pregnanolone, etiocholanolone, dihydrotestosterone, and estradiol.

Follicles were thawed, yolks were separated from the follicular membranes, and diluted with water (1:3) by vortexing with glass beads. From the resulting mixture, 200 mg was used for steroid extractions for mass spectrometric analysis by liquid chromatography combined with tandem mass spectrometry (LC-MS/MS) and 600 mg by gas chromatography combined with tandem mass spectrometry (GC-MS/MS). From entire egg homogenates (except shells), 300 mg was used for LC-MS/MS and 600 mg for GC-MS/MS. Methanol was used as organic solvent for the extractions. Details on extraction and mass spectrometry procedures can be found in the supplementary information.

### Effect of maternal enzymes in the follicular yolk on steroid metabolism

To test whether the change in yolk hormone (progesterone, androstenedione, and testosterone) concentrations between ovulation and oviposition is due to maternal enzymes in the yolk, follicular yolks were thawed and incubated at 41.4 °C for 48 hours, which mimics the conditions in the oviduct during egg development until oviposition. These follicles were not the exact same set of follicles as used for comparison with eggs as the latter had to be weight matched with the first follicles. One freeze thaw cycle is a common procedure to test the enzymatic activity of frozen tissues (e.g.^22^) and we did not expect that to affect the activity of yolk enzymes. Follicular yolks excluded extra-embryonic membranes as they were collected prior to fertilization. Aliquots of 210 mg yolk diluted with water (1:3) from seven second follicles were added with either 23.4 µl of Proteinase-K (final concentration of 2.0 mg/ml) or buffer, and incubated in a shaker at 37 °C for 3 hours with gentle mixing (250 rpm), followed by 48-hours incubation at 41.4 °C. Conditions for use of proteinase-K were optimized by testing the enzyme activity in a pilot experiment at three different final concentrations (0.5, 1.0, and 2.0 mg/ml) and two different temperatures (37 °C and 50 °C) and were found to be optimum at 37 °C at a final concentration of 2 mg/ml for 3 hours of incubation. Proteinase-K is a protein-digesting enzyme, thus if the change in hormone concentration was due to maternal enzymes there should be no change in proteinase-K treated samples, whereas it should be significantly different than non-treated samples. Target steroids were extracted using 200 mg samples from the incubation mixture while 12 µl sample aliquots were stored for protein analysis on Tris-Glycine gel. The effect of treatment on protein degradation was visualized on gels by fractionating the samples in three parts- the hydrophobic fraction, the hydrophilic fraction, and the total^23^. For the hydrophobic and hydrophilic fractions, 3 µl of sample was washed with 72 µl of acidic water (pH 5.0) by shaking gently for one hour at 4 °C. The resulting mixture was centrifuged at 12,000xg for 10 minutes at 4 °C. The pellet and the vacuum-dried supernatant were re-suspended separately in 30 µl of SDS gel loading buffer, of which 5 µl was loaded on 12% Tris-Glycine gel. To visualize the entire protein fraction, approximately 1 µl of sample was directly re-suspended in 30 µl of SDS gel loading buffer, of which 5 µl was loaded on the gel.

### Statistical analyses

All the data were analysed using IBM SPSS software, version 23.

- Comparison of follicles and eggs (Fig. 2; Table 1): data were analysed using the weighted least square method (so as not to assume equal variances across groups) in general linear models taking egg laying order and time (representing follicle and egg stages) and their interaction as fixed factors. As first follicles, second follicles, and eggs were obtained from separate females (thus, three out of four groups were independent), and since all the data were analysed in same statistical model, samples were treated as independent. Most of the residuals from the statistical model were normally distributed for all analysed steroids except estradiol and conjugated estrone. Therefore, the statistical outcomes for these two compounds were further verified and confirmed by Gamma distribution models. The standardized effect estimates were obtained using linear regression. Out of the total 320 data points, 7 data points with extreme values were excluded as outliers.
- Effect of maternal enzymes in the follicular yolk (Fig. 4): out of the three hormones analysed for the effect of maternal enzymes in the yolk on their metabolism, only androstenedione data was normally distributed and hence the difference between proteinase-K and control treatment analysed by paired t-tests. The other two hormones – progesterone and testosterone, were analysed by ‘Related Samples Wilcoxon Signed Rank’ tests. One sample being an outlier was excluded from statistical analysis of testosterone. The effect sizes (Supplementary Table 2) were calculated after Nakagawa and Cuthill^24^.

### Data availability

The dataset supporting this article can be accessed as supplementary dataset file.

### Ethics

All the animal research was conducted according to the established guidelines and regulations of the animal welfare committee of the University of Groningen, and all relevant procedures were approved by the committee under the license 6835B.

## Electronic supplementary material

Supplementary Information

Supplementary Dataset
