# Supplementary material for: Gonadal steroid levels in rock pigeon eggs do not represent adequately maternal allocation
Source: Sci Rep. 2018 Jul 25;8:11213. doi: 10.1038/s41598-018-29478-4 (PMC6060151; doi:10.1038/s41598-018-29478-4)
Supplement: Supplementary file 1 — Supplementary Information [file 41598_2018_29478_MOESM1_ESM.doc]

**Title**

Gonadal steroid levels in rock pigeon eggs do not represent adequately maternal allocation

**Authors**

Neeraj Kumar^1,3*^, Martijn van Faassen^2^, Bonnie de Vries^1^, Ido Kema^2^, Manfred Gahr^3^, Ton G.G. Groothuis^1^

*^1^Behavioural Biology, Groningen Institute for Evolutionary Life Sciences, University of Groningen, the Netherlands*

*^2^Laboratory Medicine, University Medical Center Groningen, University of Groningen, the Netherlands*

*^3^Behavioural Neurobiology, Max Planck Institute for Ornithology, Seewiesen, Germany*

*To whom correspondence should be addressed.

e-mail: neeraj.bioscience@gmail.com

**Supplementary information**

**Steroid extraction**

To each sample either 50 µl (mixture of ^13^C_3_ labelled progesterone, 17-hydroxyprogesterone, androstenedione, and testosterone in 50% methanol); or 200 µl (^2^H_5_ labelled etiocholanolone in pure methanol) of an internal standard was added and left for one hour at room temperature for equilibration. Internal standards for estrone and estradiol (^13^C_3_ labelled) were added after the extractions. Each sample was extracted twice in 1 ml methanol by vortexing, followed by centrifugation at 12000xg for 10 minutes at room temperature. The supernatant was transferred to tubes containing 200 mg of solid ZnCl_2_ for lipid precipitation^1^. The total volume of the combined supernatants was made to 4 ml by adding 2 ml methanol, and centrifuged at 12000xg for 10 minutes at 4°C. The supernatant was dried under nitrogen gas in a waterbath at 50°C, re-suspended in 1 ml methanol, centrifuged at 12000xg for 10 minutes at room temperature, followed by addition of 1.8 ml water to the supernatant. This mixture was centrifuged at 12000xg for 10 minutes at 4°C. The supernatant was loaded on C-18 SPE columns (3 ml, 500 mg, Grace Inc.) pre-equilibrated with 3 ml of methanol, followed by 3 ml of water. After collecting flow through, columns were washed with 3 ml water, and then eluted with 2 ml methanol. The eluent was divided in two equal parts, one part was analyzed without hydrolysis and the other part after hydrolysis, the difference between the two representing the conjugated steroids as hydrolysis converts conjugated compounds to their free forms (e.g.^2^).

(a) Sample preparation without hydrolysis: 1 ml eluent was dried under vacuum, re-suspended in methanol, followed by addition of water to make a final concentration of 30% methanol.

(b) Sample preparation with hydrolysis: 1 ml eluent was dried under vacuum, and re-suspended in 2 ml acetate buffer (0.5 M sodium acetate with 15 g/l sodium ascorbate, pH 4.8). 100 µl of Helix Pomatia (Brunschwig Chemie) was added, vortexed, and incubated at 46°C for 2 hours. The hydrolyzed samples were cooled at room temperature and purified on HLB SPE columns (3ml, Waters Inc) pre-equilibrated with 2 ml of methanol, followed by 2 ml of water. After collecting flow through, columns were washed with 2 ml water, and then eluted with 2 ml methanol. The eluent was dried under vacuum, re-suspended in methanol, followed by addition of water to make a final concentration of 30% methanol.

**Mass Spectrometry**

**(a) LC-MS/MS**

All extracts were analyzed with a XEVO TQ-S tandem mass spectrometer (Waters Corp.), equipped with an Online SPE Manager and ACQUITY UPLC system (Waters Corp.). The UPLC flow rate was set at 0.4 ml/min using 10 mM ammonium acetate, 0.1% formic acid in water and methanol (containing 0.1% formic acid) as mobile phases A and B respectively. The analysis of estradiol and estrone consisted of 0.2 mM ammonium fluoride in 10 % methanol in water and 0.2 mM ammonium fluoride in methanol as mobile phase A and B respectively. For each extract, 40 µl sample was injected for extraction on XBridge C8 cartridge and chromatographic separation was performed on a Kinetex C18 column (2.1 x 100 mm, 2.6 µm). The mass spectrometer was operated under electrospray ionization mode with following operating conditions: cone voltage of 30 V, desolvation temperature of 600°C and source temperature of 150°C, collision energy between 15-40 eV optimized for different analytes. Quantitative calibration was performed by using a calibration curve using the internal standards for each of the analyte. The analysis was performed by monitoring two mass transitions for each analyte. The monitored multiple reaction monitoring (MRM) transitions (m/z) are shown in the Supplementary Table 1. The quantification limits were 0.01-0.05 nmol/L, except for dehydroepiandrosterone (2.0 nmol/L) and dihydrotestosterone (0.1 nmol/L).

**(b) GC-MS/MS**

Etiocholanolone and pregnanolone were measured using gas chromatography combined with tandem mass spectrometry (GC-MS/MS)^3^. The steroids were extracted using the same procedure as for LC-MS/MS except that HLB cartridges (3 ml, 60 mg) were used for cleanup before hydrolysis, instead of C-18. Steroids were derivatized using 150 µl of methoxyamine (stock solution of 1 g methoxyamine.HCl in 50 ml pyridine) by incubating samples at 80°C for 1 hour. After evaporating the solvent at room temperature under nitrogen gas, samples were incubated with 200 µl N-trimethyl silyl imidazole overnight. Derivatized samples were washed with 4 ml n-heptane in 3 ml of 0.1 M HCl by vortexing. Upper heptane layer was collected, first by centrifuging for 4 minutes at 1200xg, and then cryo-phase separation by incubating samples for 1 minute at -45°C. This upper n-heptane layer was washed with water, and then evaporated and re-suspended in 200 µl n-heptane.

Steroids were chromatographically separated on a J&W CP-Sil 5 CB column (15 m x 250 μm x 0.25 μm). A 7890A GC with 7000 Triple Quadrupole Detector (Agilent) was used for separation and detection using electron impact and multiple reaction monitoring. Nitrogen was used as collision gas (flow 1.5 mL/min), Helium as quench gas (flow 2.25 mL/min) and carrier gas (2 mL/min). For each sample 5 µl was injected at 65°C, with the MS source at 270°C and both quadrupoles at 150°C. Chromatography was performed using a temperature program for optimal separation: 1 min 50°C, ramp 50°C/min until 200°C, and finally ramp 2.5°C/min until 230°C. Electron impact was performed at 70 eV. The monitored MRM transitions (m/z) are shown in the Supplementary Table 1. The quantification limit for etiocholanolone was 1.0 nmol/L, and for pregnanolone 10.0 nmol/L.

**References**

1. Wang, Q., Zhang, A., Pan, X. & Chen, L. Simultaneous determination of sex hormones in egg products by ZnCl2 depositing lipid, solid-phase extraction and ultra performance liquid chromatography/electrospray ionization tandem mass spectrometry. *Anal. Chim. Acta* **678,** 108–16 (2010).

2. Mi, X. *et al.* Quantitative determination of 26 steroids in eggs from various species using liquid chromatography-triple quadrupole-mass spectrometry. *J. Chromatogr. a* **1356,** 54–63 (2014).

3. Jong, W. H. A. D. *et al.* Determination of reference intervals for urinary steroid profiling using a newly validated GC-MS/MS method. *Clin. Chem. Lab. Med.* **56,** 103–112 (2017).

**Figure S1**


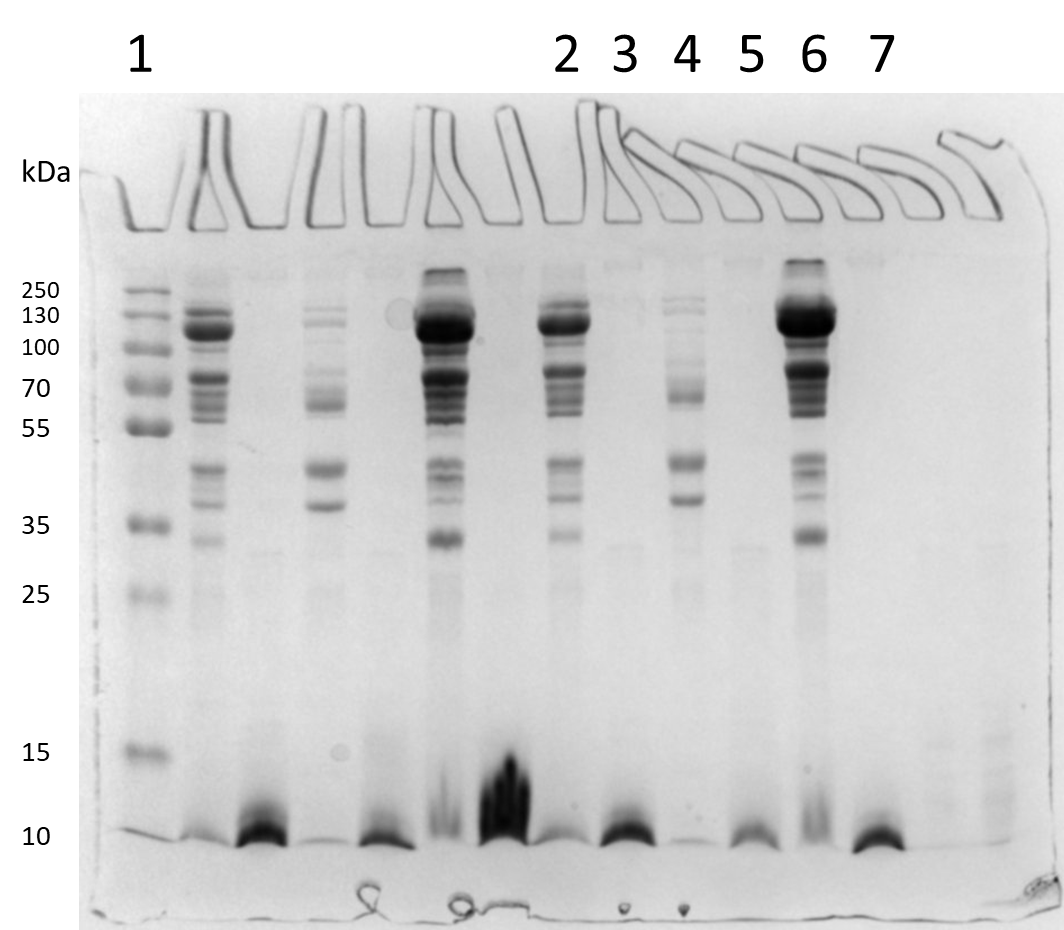


**Figure S1.** The complete gel from which lanes 1 and 2-7 were cropped in Fig. 3, corresponding to the conditions which were used to test the effect of maternal enzymes in follicular yolk on steroid metabolism.

**Supplementary Table 1.** Multiple Reaction Monitoring (MRM) transitions.

| **Compound** | **m/z** | **Cone voltage (V)** | **Collision energy (eV)** |
| --- | --- | --- | --- |
| P4 - (QN) | 315 > 97 | 30 | 40 |
| P4 - (QL) | 315 > 109 | 30 | 40 |
| P4 - 13C3 (QN) | 318 > 100 | 30 | 40 |
| P4 - 13C3 (QL) | 318 > 112 | 30 | 40 |
| 17-OH-P4 - (QN) | 331 > 97 | 30 | 25 |
| 17-OH-P4 - (QL) | 331 > 109 | 30 | 28 |
| 17-OH-P4 - 13C3 - (QN) | 334 > 100 | 30 | 25 |
| 17-OH-P4 - 13C3- (QL) | 334 > 112 | 30 | 28 |
| DHEA - (QN) | 253 > 197 | 30 | 22 |
| DHEA - (QL) | 271 > 213 | 30 | 15 |
| A4 - (QN) | 287 > 97 | 30 | 35 |
| A4 - (QL) | 287 > 109 | 30 | 35 |
| A4 - 13C3 (QN) | 290 > 100 | 30 | 35 |
| A4 - 13C3 (QL) | 290> 112 | 30 | 35 |
| T - (QN) | 289 > 97 | 30 | 35 |
| T - (QL) | 289 > 109 | 30 | 35 |
| T - 13C3 (QN) | 292 >100 | 30 | 35 |
| T - 13C3(QL) | 292 > 112 | 30 | 35 |
| DHT - (QN) | 291> 159 | 30 | 20 |
| DHT - (QL) | 291 > 255 | 30 | 15 |
| DHT - 13C3 (QN) | 294 > 162 | 30 | 20 |
| DHT - 13C3 (QL) | 294 > 258 | 30 | 15 |
| E1 - (QN) | 269 > 145 | 30 | 40 |
| E1 - (QL) | 269 > 159 | 30 | 40 |
| E1 - 13C3 (QN) | 272 > 148 | 30 | 40 |
| E1 - 13C3 (QL) | 272 > 162 | 30 | 40 |
| E2 - (QN) | 271 > 145 | 30 | 40 |
| E2 - (QL) | 271 > 183 | 30 | 40 |
| E2 - 13C3 (QN) | 274 > 148 | 30 | 40 |
| E2 - 13C3 (QL) | 274 > 186 | 30 | 40 |
| P | 388 > 298 | - | 10 |
| E | 360 > 270 | - | 5 |

m/z, mass-to-charge ratio; V, volt; eV: electron-volt; P4, progesterone; QN, quantifier; QL, qualifier; 17-OH-P4, 17-hydroxyprogesterone; DHEA, dehydroepiandrosterone; A4, androstenedione; T, testosterone; DHT, dihydrotestosterone; E1, estrone; E2, estradiol; P, pregnanolone; E, etiocholanolone (more detailed information can be found for P and E in ref 3 in the supplementary information).

**Supplementary Table 2. Descriptive statistics, p-values, and effect size for metabolism of follicular yolk hormones corresponding to Fig. 4. Mean values are given for androstenedione (A4) as the data was normally distributed, and median values are given for testosterone (T) and progesterone (P4) as the data were not normally distributed for these two hormones.**

| **Hormone** | **Comparison** | **Mean (for A4) and median (for T and P4) values (ng)** | **p-value** | **Effect Size (Cohen’s d)** |
| --- | --- | --- | --- | --- |
| Progesterone | 0 hrs: 48 hrs (no treatment) | 0 hrs: 2379.80  48 hrs: 2220.89 | 0.499 | 0.03 |
|  | 0 hrs: 48 hrs (Proteinase-K treatment) | 0 hrs: 2379.80  48 hrs: 2124.59 | 0.310 | 0.02 |
|  | interaction (48 hrs Proteinase-K treatment : 48 hrs no treatment) | 48 hrs Proteinase-K treatment: 2124.59  48 hrs no treatment: 2220.89 | 0.499 | 0.01 |
| Androstenedione | 0 hrs: 48 hrs (no treatment) | 0 hrs: 385.82  48 hrs: 336.25 | **0.004** | 0.32 |
|  | 0 hrs: 48 hrs (Proteinase-K treatment) | 0 hrs: 385.82  48 hrs: 336.26 | **0.002** | 0.32 |
|  | interaction (48 hrs Proteinase-K treatment : 48 hrs no treatment) | 48 hrs Proteinase-K treatment: 336.26  48 hrs no treatment: 336.25 | 0.999 | 0.00 |
| Testosterone | 0 hrs: 48 hrs (no treatment) | 0 hrs: 8.42  48 hrs: 8.31 | **0.046** | 0.13 |
|  | 0 hrs: 48 hrs (Proteinase-K treatment) | 0 hrs: 8.42  48 hrs: 8.23 | **0.028** | 0.15 |
|  | interaction (48 hrs Proteinase-K treatment : 48 hrs no treatment) | 48 hrs Proteinase-K treatment: 8.23  48 hrs no treatment: 8.31 | 0.249 | 0.01 |

**Supplementary Table 3.** Effect sizes (Cohen’s d) for comparisons of second follicles with second eggs for levels of progesterone, androstenedione, and testosterone.

| **Hormone** | **Cohen’s d** |
| --- | --- |
| Progesterone | 3.19 |
| Androstenedione | 1.14 |
| Testosterone | 1.41 |

**Supplementary Table 4.** Statistical parameters for comparisons of first and second follicles (column 3), and first and second eggs (column 4).

| **Steroid** | **Statistical parameters** | **Laying order**  **(first vs second)** | |
| --- | --- | --- | --- |
|  | | for pre-ovulatory follicles | for eggs |
| Progesterone | Coefficient (beta) | 0.138 | 0.450 |
|  | Std Error | 0.148 | 0.330 |
|  | p value | 0.341 | 0.218 |
| 17-Hydroxyprogesterone | Coefficient (beta) | 1.539 | 0.901 |
|  | Std Error | 0.323 | 0.373 |
|  | p value | **<0.001** | **0.032** |
| Androstenedione | Coefficient (beta) | 1.771 | 1.016 |
|  | Std Error | 0.281 | 0.197 |
|  | p value | **<0.001** | **<0.001** |
| Testosterone | Coefficient (beta) | 1.736 | 0.930 |
|  | Std Error | 0.264 | 0.204 |
|  | p value | **<0.001** | **<0.001** |
| Estrone | Coefficient (beta) | 0.228 | 0.459 |
|  | Std Error | 0.490 | 0.222 |
|  | p value | 0.651 | 0.053 |
| Estradiol | Coefficient (beta) | 0.475 | 0.075 |
|  | Std Error | 0.479 | 0.296 |
|  | p value | 0.367 | 0.745 |
| Conjugated testosterone | Coefficient (beta) | 0.844 | -0.126 |
|  | Std Error | 0.484 | 0.102 |
|  | p value | 0.097 | 0.233 |
| Conjugated estrone | Coefficient (beta) | -0.819 | 0.773 |
|  | Std Error | 0.361 | 0.565 |
|  | p value | **0.032** | 0.202 |
